# Calorimetric and Dielectric Study of Renewable Poly(hexylene 2,5-furan-dicarboxylate)-Based Nanocomposites In Situ Filled with Small Amounts of Graphene Platelets and Silica Nanoparticles

**DOI:** 10.3390/polym12061239

**Published:** 2020-05-29

**Authors:** Olawale Monsur Sanusi, Lazaros Papadopoulos, Panagiotis A. Klonos, Zoi Terzopoulou, Nourredine Aït Hocine, Abdelkibir Benelfellah, George Z. Papageorgiou, Apostolos Kyritsis, Dimitrios N. Bikiaris

**Affiliations:** 1 INSA CVL, Univ. Tours, Univ. Orléans, LaMé, 3 Rue de la Chocolaterie, CS 23410, CEDEX 41034 Blois, France; olawale.sanusi@insa-cvl.fr (O.M.S.); nourredine.aithocine@insa-cvl.fr (N.A.H.); abdelkibir.benelfellah@ipsa.fr (A.B.); 2 Department of Chemistry, Laboratory of Polymer Chemistry and Technology, Aristotle University of Thessaloniki, GR-541 24 Thessaloniki, Greece; lazaros.geo.papadopoulos@gmail.com (L.P.); terzoe@gmail.com (Z.T.); 3 Department of Physics, National Technical University of Athens, Zografou Campus, 15780 Athens, Greece; akyrits@central.ntua.gr; 4 DRII, IPSA, 63 Boulevard de Brandebourg, 94200 Ivry-Sur-Seine, France; 5 Laboratory of Industrial and Food chemistry, Chemistry Department, University of Ioannina, 45110 Ioannina, Greece; gzpap@cc.uoi.gr

**Keywords:** poly(hexylene 2,5-furan-dicarboxylate), FDCA based polyesters, furan-based nanocomposites, polymer nanocomposites, graphene, crystallization, dielectric spectroscopy, molecular dynamics

## Abstract

Poly(hexylene 2,5 furan-dicarboxylate) (PHF) is a relatively new biobased polyester prepared from renewable resources, which is targeted for use in food packaging applications, owing to its great mechanical and gas barrier performance. Since both properties are strongly connected to crystallinity, the latter is enhanced here by the in situ introduction in PHF of graphene nanoplatelets and fumed silica nanoparticles, as well as mixtures of both, at low amounts. For this investigation, we employed Fourier transform infrared spectroscopy (FTIR), differential scanning calorimetry (DSC), X-ray diffraction (XRD) and dielectric spectroscopy (BDS). The fillers were found to improve crystallization in both the rate (increasing *T*_c_) and fraction (CF), which was rationalized via the concept of fillers acting as crystallization agents. This action was found stronger in the case of graphene as compared to silica. BDS allowed the detection of local and segmental dynamics, in particular in PHF for the first time. The glass transition dynamics in both BDS (α relaxation) and DSC (*T*_g_) are mainly dominated by the relatively high CF, whereas in the PHF filled uniquely with silica strong spatial confinement effects due to crystals were revealed. Finally, all samples demonstrated the segmental-like dynamics above *T*_g_, which screens the global chain dynamics (normal mode).

## 1. Introduction

Polymeric materials are used in a wide range of applications in industry, academia, and everyday life. This is mainly due to the combination of low production cost with good performance in wanted properties. During the past decades, polymer nanocomposites (PNCs), polymers reinforced with properly chosen nano-inclusions [1,2,3,4], have attracted serious attention and have furthermore opened the way for novel applications. PNCs demonstrate tremendously improved properties [5,6] compared to unfilled polymers and macro-composites, owing mainly to the large surface to volume ratio of the used nano-inclusions [5,7,8]. The nano-inclusions (fillers) can be categorized based on their dimensionality to platelets (such as nanoclays and graphene) [9,10], spherical particles (such as silica) [11], and nanotubes (e.g., multi/single walled carbon nanotubes, MWCNT/SWCNT) [12].

Quite recently, the world has developed increasing interest in environmental concerns, including the use of commodity plastics. Plastics are prepared from finite raw sources and do not have eco-friendly properties due to their extremely slow biodegradability and expensive and hazardous recycling processes. A solution for this is being sought via the development and application of biodegradable polymers produced from renewable resources. Such polymers are, for example: poly(lactic acid) [13,14], poly(*ε*-caprolactone) [15,16], the aliphatic polyester poly(butylene succinate) [17,18], and polyvanillates [19]. In addition, biobased polyesters produced from renewable resources, e.g., from 2,5-furan-dicarboxylic acid (FDCA), form a relatively new class of materials targeted to replace their fossil-based homologues in the near future [20,21,22]. FDCA is a renewable building block derived from 5-hydroxymethylfurfural sources. It is the bio-derived homologue of terephthalic acid [23,24], a monomer widely used for the production of commodity plastics of today, as high molecular weight poly(ethylene 2,5-furan-dicarboxylate) (PEF) [25,26,27] and other poly(alkylene 2,5-furan-dicarboxylate)s. In this context, poly(hexylene 2,5-furan-dicarboxylate) (PHF) [28], the polymer of interest in the present study, is a new biobased polyester based on FDCA. These types of polyesters have already found interest [29,30] as suitable materials for packaging, since they demonstrate excellent performance regarding their gas barrier properties [31], due to their chain conformation and molecular dynamics [32], as well as due to their good mechanical properties. Furthermore, FDCA-based polyesters present an additional advantage in their semicrystalline character, which is also responsible for their good mechanical performance. In contrast to poly(propylene/butylene 2,5-furan-dicarboxylate) (PPF/PBF) [33,34] which shows poor crystallization, PHF seems to crystallize faster and in larger amounts [28].

As in the majority of semicrystalline polymers, the mechanical performance in the FDCA-based polyesters is strongly connected with the degree of crystallinity and crystal structure [34,35,36,37]. To increase crystallinity, except for modifying the polymer structure or developing special thermal treatments, another common strategy refers to the introduction in the polymer matrix of properly selected inorganic nano-inclusions [7,16,38]. This strategy proved successful in our recently published work for PPF and PBF [34,36,37]. Furthermore, we showed that even very small amounts of filler loadings (0.5, 1, 2 wt %) can impose significant improvements in the rate of crystallization of FDCA-based polyesters, in particular when this is combined with in situ PNC synthesis. These improvements were interpreted in terms of the fillers acting as additional (heterogeneous) crystallization nuclei, whereas, interestingly, the filler aspect ratio (AR) seems to be the key parameter that controls the crystallization enhancement [7,37,39,40]. 

Herein, we extend the aforementioned strategy to PHF. In particular, we investigate crystallization, structure, thermal transitions, and molecular mobility in a series of PNCs based on a PHF matrix reinforced with low amounts of graphene nanoplatelets and fumed silica nanoparticles, individually and in combination with each other. It is crucial that the nanofillers are introduced in PHF in situ, i.e., their dispersion in the polymer matrix occurs simultaneously with the polymerization. Graphene and silica were chosen from amongst previously used fillers due to the relatively high and lower AR, respectively, in addition to their different surface chemistry characteristics. It should be noted that this is not the first time that graphene has been introduced as the reinforcing mean in a biobased polymer [41,42,43]. For the scope of this investigation we employed a combination of conventional differential scanning calorimetry (DSC) and broadband dielectric spectroscopy (BDS) supplemented by X-ray diffraction (XRD) and Fourier transform infrared spectroscopy (FTIR). The molecular dynamics for neat PHF by BDS is shown here for the first time, to the best of our knowledge.

## 2. Materials and Methods 

### 2.1. Materials

**Figure 1:**
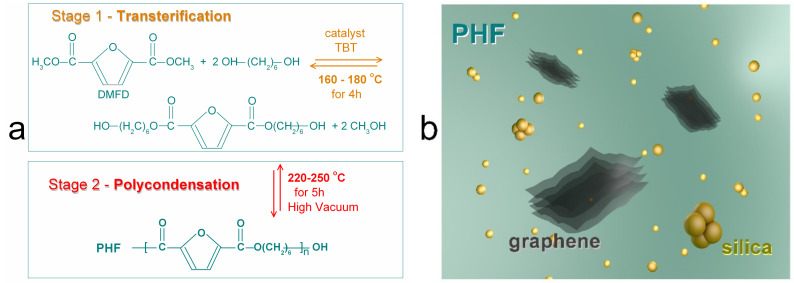
(**a**) Transterification and polycondensation stages of synthesis for Poly(hexylene 2,5 furan-dicarboxylate) (PHF). (**b**) Schematic illustration for the nanocomposites of PHF filled with graphene nanoplatelets and fumed silica spherical nanoparticles.

PHF as well its nanocomposites were synthesized by a known two-stage process involving transterification and polycondensation (Figure 1a). Details on the polymer synthesis processes can be found in previous work [28,33,44]. The necessary chemicals were all of analytical grade and had been purchased from Sigma-Aldrich Co. (Taufkirchen, Germany). For the synthesis, we used 2,5-dimethylfuran-dicarboxylate (DMFD), 1,6 hexanediol (HDO), and Tetra-tert-butyl orthotitanate (TBT) catalyst. The nanofillers were graphene nanoplatelets (XG Sciences, Lansing, MI, USA, xGnP-Grade M5, thickness 6–8 nm, size/diameter 5–25 μm, specific surface area *S*_area_ ~120–150 m^2^/g, mass density 2.2 g/cm^3^) and silica nanoparticles (SiO_2_, Aerosil^®^200, Evonik, Essen, Germany, specific surface area ~200 m^2^/g and particle size < 15 nm).

The studied materials consist of six (6) samples (Table 1, Figure 1b), the unfilled matrix **PHF**, PHF + 1.0 wt % silica nanoparticles (**P10S**), PHF + 1.0 wt % graphene nanoplatelets (**P10Gr**), and three nanocomposites of PHF filled with both graphene (at 1.0 wt %) and silica in three amounts, 0.5%, 1.0% and 2.5% (**P10Gr05S**, **P10Gr10S** and **P10Gr25S**). The final products of synthesis were thermal-pressed in a special homemade Teflon mold at 200 °C, which is above their melting temperature. Immediately after the thermal pressing, the samples were quenched by immersion into liquid nitrogen. This way the samples were formed as cylindrical disks of ~20 mm in diameter and ~1.4 mm in thickness.

### 2.2. Fourier Transform Infrared Spectroscopy (FTIR)

The FTIR measurements were performed by means of a SPECTRUM 1000 Perkin-Elmer FTIR apparatus (Waltham, MA, USA) on samples as received, granulated to fine powder, mixed with KBr and mechanically pressed to form cylindrical pellets. The wavenumber resolution for each spectrum was 2 cm^–1^, and the number of co-added scans was 16. The spectra presented below were baseline corrected and converted to absorbance mode.

### 2.3. Differential Scanning Calorimetry (DSC)

DSC was employed to study the thermal transitions, i.e., crystallization, melting and glass transition. To that aim, a TA Q200 DSC instrument (TA, New Castle, DE, USA) was employed, calibrated with sapphires for heat capacity and indium for temperature. Measurements were performed in nitrogen atmosphere of high purity (99.9995%) in the temperature range from –60 to 200 °C, on samples of ~8 mg in mass, cut from the prepared disks, closed in TA T–zero aluminum pans. All samples were subjected to a first heating scan at 10 K/min to erase their thermal history, upon which all samples showed a semicrystalline character, despite the abovementioned melt-quenching (in liquid nitrogen) process. Then, two main thermal protocols were performed: (*Scan 1*) The melted samples were cooled fast at ~95 K/min to –60 °C, held there for a period of 10 min, and were subsequently heated to 200 °C at the rate of 10 K/min. (*Scan 2*) Melted samples were cooled to –20 °C at 10 K/min, held there for 2 min, and were subsequently heated to 200 °C at 10 K/min.

The DSC results were analyzed in terms of characteristic values of crystallization/melting temperatures and enthalpies, *T*_c/m_ and Δ*H*_c/m_, glass transition temperature, *T*_g_, and further in terms of crystalline fraction (CF). For the evaluation of CF, Equation (1) was employed,

(1) $$CF_{c}=\frac{\Delta H_{c,n}}{\Delta H_{100\%,cryst}^{}}=\frac{\Delta H_{c}}{(1-w_{filler})\Delta H_{100\%,cryst}^{}}$$

where Δ*H*_c,n_ is the crystallization enthalpy, Δ*H*_c_, normalized to the polymer mass fraction (1−*w*_filler_), and Δ*H*_100%,cryst_ is the enthalpy of 100% crystalline PHF taken equal to 143 J/g based on previous work by Papageorgiou et al. [28].

### 2.4. X-ray Diffraction (XRD)

XRD was employed to study the crystalline structure, at room temperature, in the samples as received (in the form of cylindrical disks) and isothermally annealed at 100 °C for 1 h. The XRD spectra were recorded by means of a MiniFlex II XRD system (Rigaku Co., Tokyo, Japan), with Cu Ka radiation (*λ* = 0.154 nm), over the 2*θ* range from 5° to 45° with a scanning rate of 1 deg/min.

### 2.5. Broadband Dielectric Spectroscopy (BDS)

BDS was employed to study the molecular dynamics [45] of all samples as received. The complex dielectric permittivity, ε* = ε΄−i·ε΄΄, was recorded isothermally in nitrogen atmosphere (flow) as a function of frequency in the range from 10^–1^ to 10^6^ Hz and in the temperature range between –130 and 110 °C at steps of 5 and 10 K. For that, we employed a Novocontrol BDS setup (Novocontrol GmbH, Hundsangen, Germany) and in particular, an Alpha frequency response analyzer (FRA) combined with a Quatro liquid nitrogen cryosystem. The samples in thin disk form of 20 mm in diameter and ~1.4 mm in thickness were placed between finely polished brass electrodes to form an electrical capacitor, onto which an alternating voltage was applied by means of a Novocontrol BDS-1200 sample cell.

The data for the imaginary part of dielectric permittivity, *ε*΄΄ (dielectric loss) were analyzed by the critical fitting of a Havriliak-Negami, HN, model term [46] (Equation (2)) to each *ε*΄΄(*f*) peak.

(2) $$\varepsilon^{*}(f)=\varepsilon_{\infty }+\frac{\Delta \varepsilon }{{\left(1+{\left(if/f_{0}\right)}^{\alpha_{HN}}\right)}^{\beta_{HN}}}$$

In Equation (2), *f*_0_ is a characteristic frequency related to the frequency of maximum dielectric loss (*f*_max_), *ε_∞_* describes the value of the real part of dielectric permittivity, *ε′*, for *f* >> *f*_0_, Δ*ε* is the dielectric strength and *α*_HN_ and *β*_HN_ are the shape parameters of the width and symmetry, respectively, of the fitted *ε*΄΄(*f*) peak. Upon the careful analysis process, we constructed the dielectric relaxation map (or else Arrhenius plots) demonstrating the reciprocal temperature (1000/*T*) dependence of log*f*_max_ for each process (time scale). For local and non-cooperative processes the time scale obeys the Arrhenius law (Equation (3)) [45,47], namely demonstrating an activation energy independent from *T*.

(3) $$f(T)=f_{0,Arrh}\exp\left(-\frac{E_{act}}{kT}\right)$$

In Equation (3), *f_0,Arrh_* is a frequency constant and *E_act_* is the activation energy of the relaxation [47]. In the case of segmental/cooperative processes, the time scale usually follows the Vogel-Tammann-Fulcher-Hesse law (Equation (4)) [45,48,49,50], wherein *f*_0_ is a frequency constant, *T*_0_ is the Vogel temperature, and *D* is the strength parameter related to the steepness or fragility index.

(4) $$f=f_{0}\exp\left(-\frac{DT_{0}}{T-T_{0}}\right)$$

## 3. Results and Discussion

Before proceeding with the presentation and discussion of results, we should note that the relatively low amount of 1 to 3.5 wt % for such fillers, and especially graphene, along with the in situ preparation method for PNCs, were selected based on our previous experience. In similar polyesters based on FDCA and reinforced with graphene [36,37], CNTs [51], MMTs [34] at such low loadings were found to demonstrate optimum improvements in crystallization and mechanical performance in connection to good filler dispersion. In addition, out of the methods for preparation of PNCs, e.g., melt-mixing, “in situ polymerization” offers the benefit of better and more uniform filler dispersion within the polymer matrix. This is because prior to polymerization, the monomer is used to prepare a dispersion with the nanofiller. Then, the nanofiller/monomer dispersion is subjected to sonication for 30 min in order to form a uniform dispersion in HDO, prior to the reaction for the PHF synthesis. Thus, the dispersion of the fillers already exists from the beginning stage of the reaction and is sustained throughout the polymerization.

**Figure 2:**
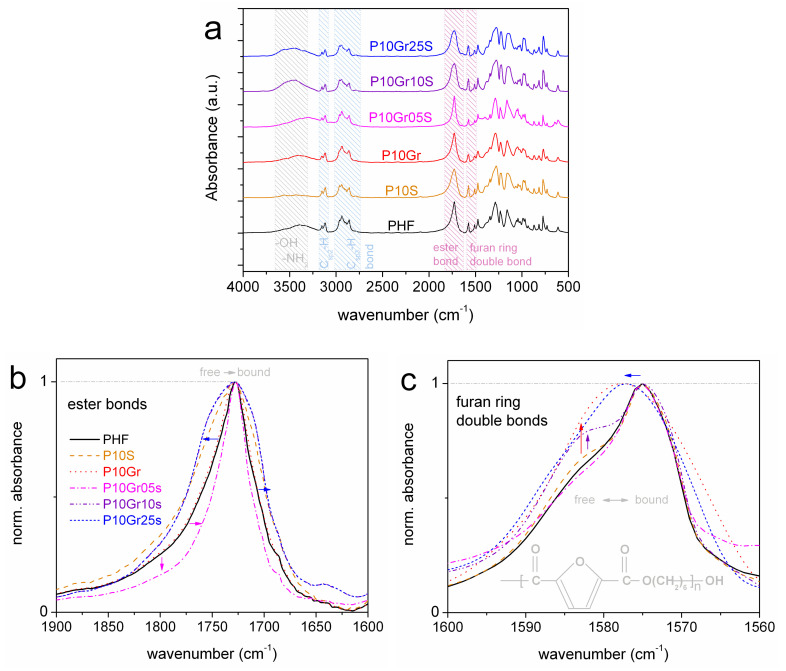
(**a**) Comparative FTIR spectra of PHF and all polymer nanocomposites (PNCs). The two distinct FTIR peaks of main interest (ester bonds and double bonds of the furan rings) are shown separately in (**b**,**c**), upon shape normalization to the peak maximum. The added arrows in (**b**,**c**) mark the effects imposed by the filler addition to PHF.

In Figure 2 we show the FTIR spectra comparatively for all samples. The results confirm the successful synthesis of the polyesters, in accordance to previous work [51,52,53]. The expected C_sp2_–H FTIR peaks are located at 3150 and 3120 cm^–1^ respectively. At 2785, 2905, and 2965 cm^–1^ the FTIR peaks of C_sp3_–H bonds of 1,6 hexanediol are recorded. At ~1725 cm^–1^ and ~1575 cm^–1^ we recorded the disturbances in the ester bonds and the furan ring double bonds, respectively. These bonds are expected to be more seriously involved within potential filler–PHF interactions [51]. More details on these regions are provided in Figure 2b,c, in addition, upon shape normalizations to each peak maximum. Therein, the aforementioned peaks do not exhibit serious disturbances, for example, migrations of the PHF peaks toward lower wavenumbers in the PNCs, which would suggest the transition from ‘free’ to ‘bound’ groups due to interactions [51,54,55]. On the contrary, we mainly record in Figure 2b,c the widening of the ester bonds peak to both directions (Figure 2b) and an average migration of the furan ring double bonds to slightly higher wavenumbers (Figure 2c). These results, thus, suggest no serious interfacial interactions between the fillers and the polymer.

**Figure 3:**
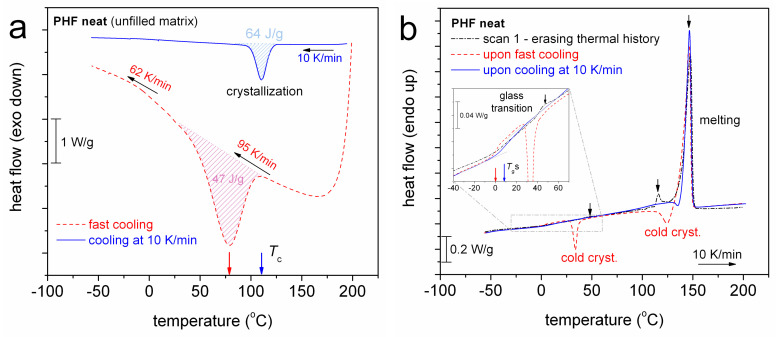
Differential scanning calorimetry (DSC) thermograms for neat PHF during (**a**) cooling at a fast (~95 K/min, scan 1) and a slower rate (10 K/min, scan 2) and (**b**) subsequent heating at 10 K/min. The main thermal events are indicated on the plots. The heat flow (in mW) has been normalized to the sample mass (in W/g). In (**b**), data of the initial run for erasing the thermal history have been included for comparison, whereas the inset plot provides more details of the glass transition region.

We come now to the results by calorimetry. In Figure 3, we follow the DSC thermograms of neat PHF and in particular the effects on crystallization between a standard cooling rate (10 K/min, scan 2) and a much faster one (~95 K/min, scan 1). For the standard cooling, *T*_c_ is 110 °C, Δ*H*_c_ = 64 J/g, *T*_g_ = 7 °C and the main melting is recorded at *T*_m_ = 146 °C. For both cooling rates, PHF demonstrates crystallization during cooling, with CF (Equation (1)) being equal to 0.45 wt and 0.33 wt for the slower and faster rate, respectively. The higher rate suppresses CF (lower crystallization enthalpy) during cooling (Figure 3a, CF and *T*_c_) and, as expected, promotes cold crystallization during heating (Figure 3b).

We notice that the samples as received, that were melted in the thermal press at 200 °C and quenched into liquid nitrogen, showed a semicrystalline character in DSC (PHF in Figure 3b) despite the exceptionally high cooling rate (in the order of 10^3^ K/min). The same was also found true in the PNCs (not shown). Thus, we conclude that by conventional techniques, we were not able to prepare amorphous PHF. This fact for PHF becomes more interesting when comparing with similar polyesters based, however, on shorter monomers e.g., PPF (propylene–) [36,51] and PBF (butylene–) [34], which for cooling rates above 60–70 K/min (cooling from the melt) could remain amorphous. Thus, with the increase of the methylene sequences in the monomer (by only two), nucleation is significantly enhanced and thus the crystallization is facilitated, which is both interesting and wanted from the processing point of view.

**Figure 4:**
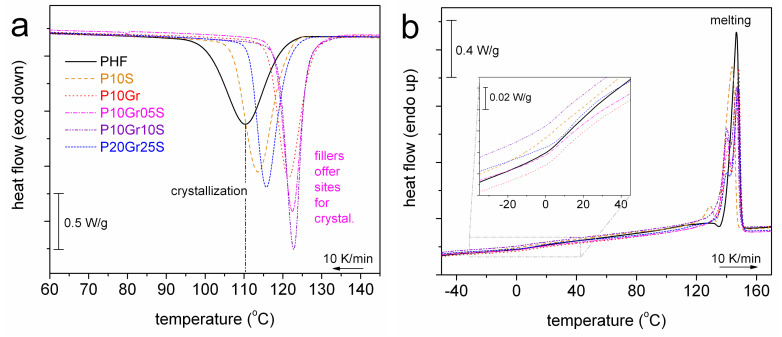
Comparative DSC thermograms for all samples of scan 2, (**a**) during cooling at 10 K/min and (**b**) during the subsequent heating at 10 K/min. The heat flow has been normalized to the sample mass. The inset in (**b**) focuses on the temperature range of the glass transition region.

We proceed to the comparison between unfilled PHF and the PNCs. The comparison is based on results of scan 2 (cooling at 10 K/min). Figure 4a shows the evolution of crystallization. It is clear, already from a glance of Figure 4a, that the filler addition imposes an increase in *T*_c_ (Table 1), independently from the filler type. Regarding heating, in Figure 4b melting of crystals exhibits only qualitative changes in the shape of the peak with additional contributions at lower temperatures (*T*_m1_ 126–140 °C in Table 1) and the main/final peak demonstrating unremarkable changes (*T*_m2_ 146–148 °C in Table 1). Glass transition is in general weak (small Δ*c*_p_) and smooth, which is most probably related to the high CF (≥0.45 wt, Table 1). This is also the reason for the insignificant alternations in *T*_g_ (Table 1). An exception to that is the surprisingly lower *T*_g_ = 1 °C in P10S, when CF in this sample is the highest (0.49 wt) among all samples. We will comment further on this interesting point later, in the light of results by BDS on molecular dynamics.

**Figure 5:**
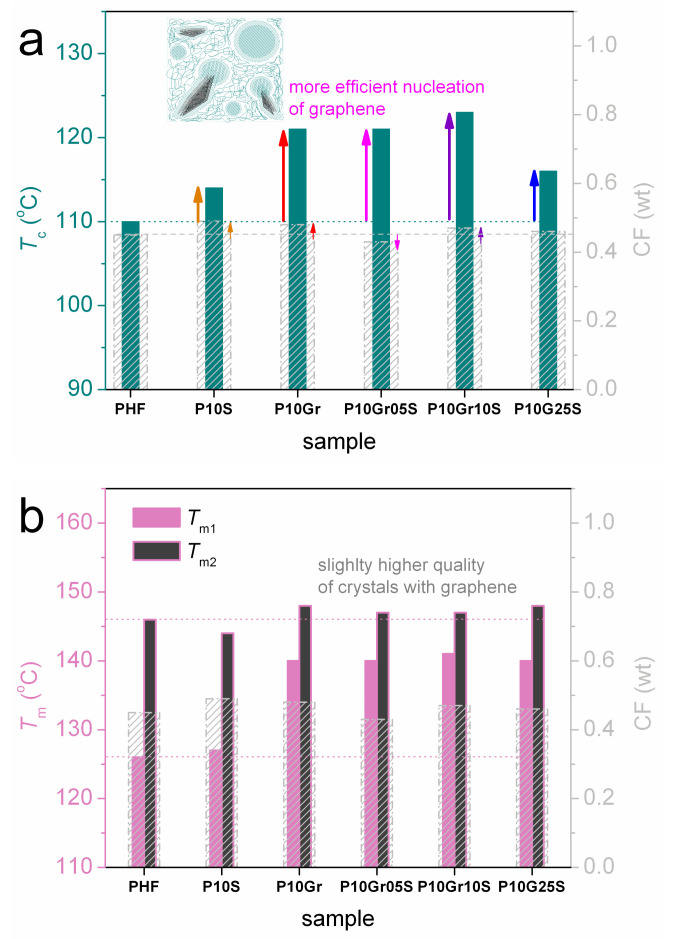
(**a**) Crystallization temperature, *T*_c_, and (**b**) melting temperatures *T*_m1,2_ against the crystalline fraction, CF, in the form of column diagrams for all samples. The vertical arrows added in (**a**) mark the effects imposed by the filler addition, whereas the inset scheme provides a simplified schematic view on the estimated filler–polymer crystals distribution.

Obviously, the most clear and interesting results of DSC are in regards to crystallization. In Figure 5 we compare for all samples the results on CF with those of *T*_c_ (Figure 5a) and *T*_m_ (Figure 5b). In almost all PNCs CF is slightly increased, whereas *T*_c_ strongly increases, from 110 °C up to 123 °C (Table 1). This increase can be understood in terms of the fillers offering sites for additional crystallization (inset scheme to Figure 5a) and acting, in this way, as additional crystallization nuclei, as has been found in different [7,38,39,55,56,57,58,59] and similar polymers [34,36,37]. In Figure 5a, the stronger increase in nucleation is recorded in the graphene containing PNCs (P10Gr, P10Gr05S, P10Gr10S), while the effect of silica is significantly more limited (P10S, P10Gr25S). One way to rationalize this difference is via the larger aspect ratio of graphene in comparison to silica [7,39,40].

These effects in *T*_c_ and CF by the filler addition provide significant although indirect indications for good filler dispersion in the PHF matrix, in relation also to the in situ preparation method discussed previously. Finally, the graphene containing PNCs exhibits slightly higher *T*_m_ values, more pronounced for *T*_m2_ (Figure 5b), which could reflect a different quality of crystals, namely in thickness of lamellar packing and/or, in general, the size of the additional crystallites.

**Figure 6:**
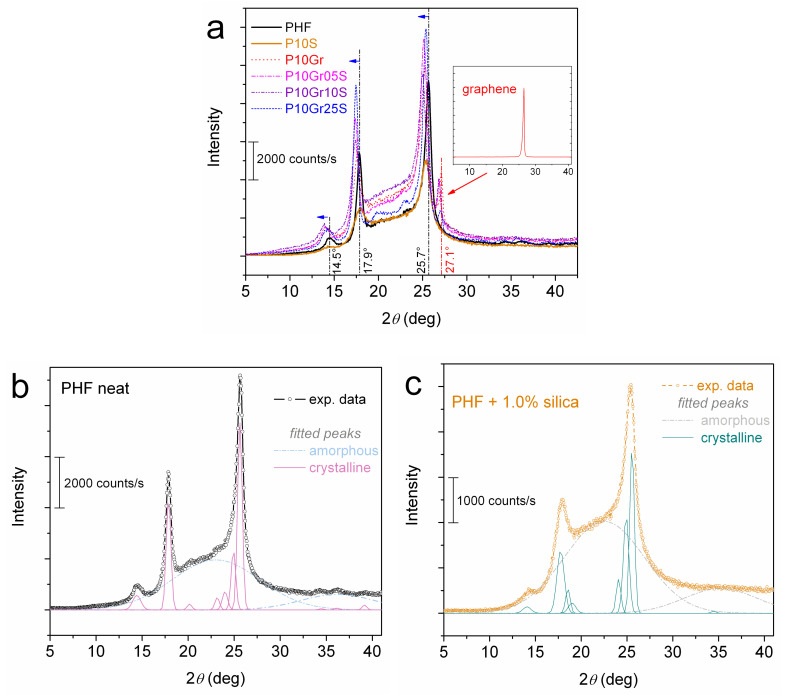
(**a**) XRD spectra shown comparatively for all samples. The added vertical lines and arrows in (**a**) are used to denote changes in the recorded crystalline peaks between the PNCs and the unfilled matrix while the inset is the XRD result for initial graphene. (**b**,**c**) show examples of fitting of the XRD spectra in terms of Gaussian peaks for neat PHF and P10S, respectively.

Support for the latter proposed difference in the semicrystalline morphology is provided by XRD results shown in Figure 6. Therein, the crystalline diffraction peaks are almost at the same 2*θ* positions in PHF and P10S. On the other hand, in all the graphene containing PNCs, the crystalline peaks are located at lower 2*θ* positions, i.e., indicating increased lamellae thickness. However, we note that the number of crystalline peaks is almost the same for all samples. More evidence for the expected alternations in the semicrystalline morphology, especially at a large scale, could be provided by polarized optical microscopy (POM) measurements [55,60].

**Figure 7:**
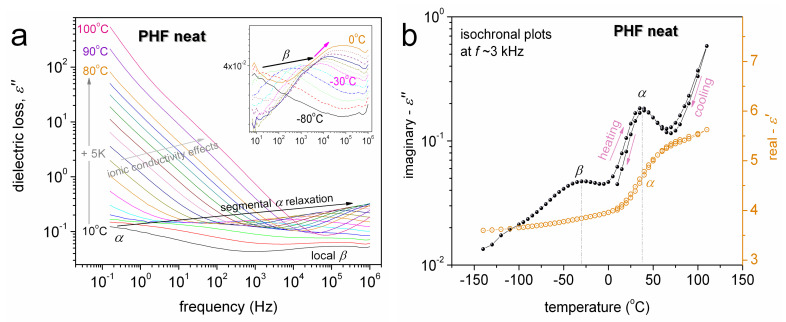
(**a**) Isothermal broadband dielectric spectroscopy (BDS) plots of the imaginary part of dielectric permittivity (dielectric loss), *ε΄΄*, against frequency for neat PHF. The main relaxation processes recorded are indicated on the plots. (**b**) shows isochronal BDS plots of *ε΄΄* comparatively with those of the real part of dielectric permittivity (dielectric storage), *ε΄*, against temperature during heating and subsequent cooling (arrows) at the selected frequency of 3 kHz.

We proceed with molecular dynamics by BDS. BDS results are discussed here mainly in terms of *ε΄΄* against frequency (isothermal plots) and temperature (isochronal plots). The isochronal plots, in particular, provide an easier comparison with results by calorimetry. Figure 7a shows the raw BDS isothermal data for neat PHF, while in Figure 7b selected *ε΄΄* data of Figure 7a, at *f*~3 kHz, were replotted and shown comparatively with *ε΄*. Two relaxation processes can be seen therein, the local *β* at lower temperatures and segmental *α* at *T* > *T*_g_.

The local *β* process has been recorded in similar polyesters based on furan 2,5-furan-dicarboxylate [34,37,61] and has been proposed to originate from crankshaft motions of the molecular group related to the chemical link between the aromatic ring and the ester carbon.

*α* is widely considered the dielectric analogue of the glass transition and thus is also called the ‘glass transition dynamics’ [45]. *α* is recorded as a peak in *ε΄΄* and as an increasing *ε΄* step with temperature (Figure 7b). In Figure 7a, *α* is located at elevated temperatures (~35 °C) as compared to *T*_g_ (7 °C) due to the selected frequency of 3 kHz, which is higher than the equivalent frequency of DSC (1.6 mHz) [45].

**Figure 8:**
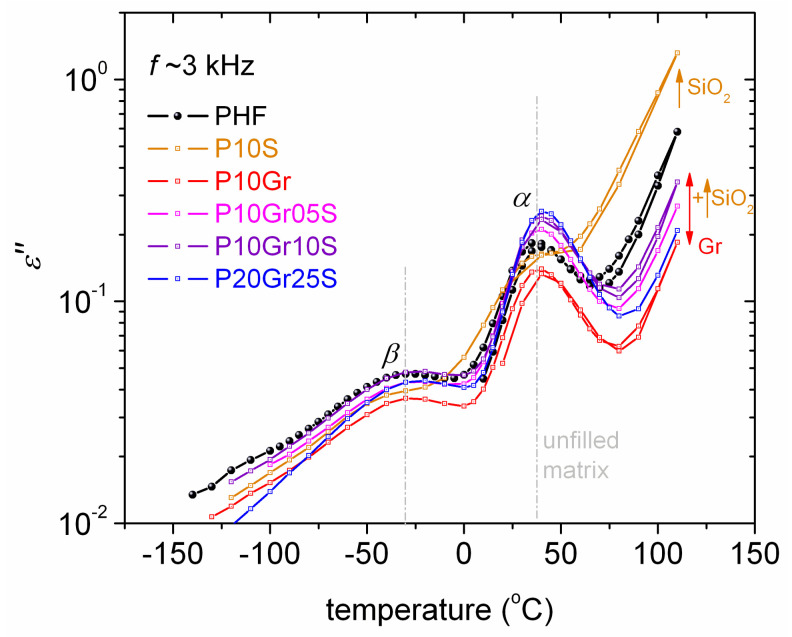
Comparative isochronal plots of *ε΄΄*, (dielectric loss) against temperature 3 kHz for all samples. The recorded relaxation processes are indicated on the plot.

In Figure 8 we present the isochronal plots at 3 kHz comparatively for all samples. The first impression given by Figure 8 is that both *β* and *α* are located at quite similar temperature positions, and the main differences arise from their magnitudes (strengths). At the higher temperatures, namely above 75 °C, the strongly rising signal is due to ionic conductivity phenomena [45,62] as the polymer matrix is at the rubbery state and ion transport is favored through the polymer volume. 

From the values of *ε΄΄*, *ε΄,* and conductivity (*σ΄*, not shown) at the highest temperatures, which are in general low and lay at similar ranges for all samples, it is clear that in all cases we deal with electronically isolating materials. Therefore, we conclude that the graphene fillers (electron conductors) at the 1 wt % loadings do not percolate throughout the PNC volume, which is desired for the envisaged applications and from the point of view of the production cost. 

**Figure 9:**
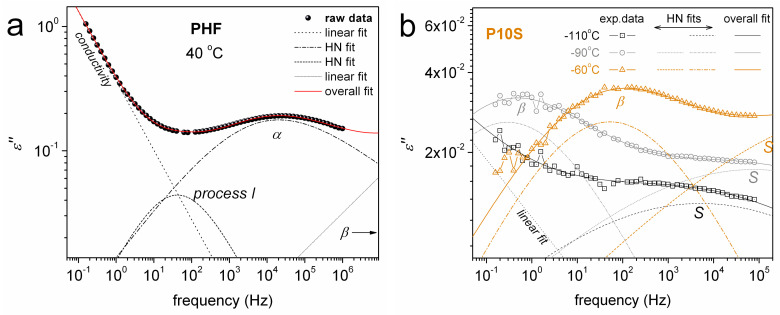
Examples of fitting of the *ε΄΄*(*f*) data in terms of HN model (Equation (2)) functions for the relaxation peaks, (**a**) neat PHF at 40 °C and (**b**) P10S at –110, –90 and –60 °C.

Next, to better evaluate the dynamics of the recorded processes, we performed analysis of the *ε*΄΄(*f*) spectra by fitting to each peak a HN term (Equation (2), Section 2.5). Paradigms of this analysis are presented in Figure 9, at the examples of neat PHF at 40 °C (Figure 9a) and P10S at –110, –90 and –60 °C (Figure 9b). Interestingly, the critical analysis revealed additional weak relaxation processes, which could not be identified directly with the naked eye from the raw data. First, next to *α* relaxation at slightly lower frequencies the additional *process Ι* (Figure 9a) could be resolved for all samples except P10S (PHF with 1 wt % silica). Then, in P10S the additional *S* relaxation was identified at higher frequencies/lower temperatures than *β* relaxation (Figure 9b).

**Figure 10:**
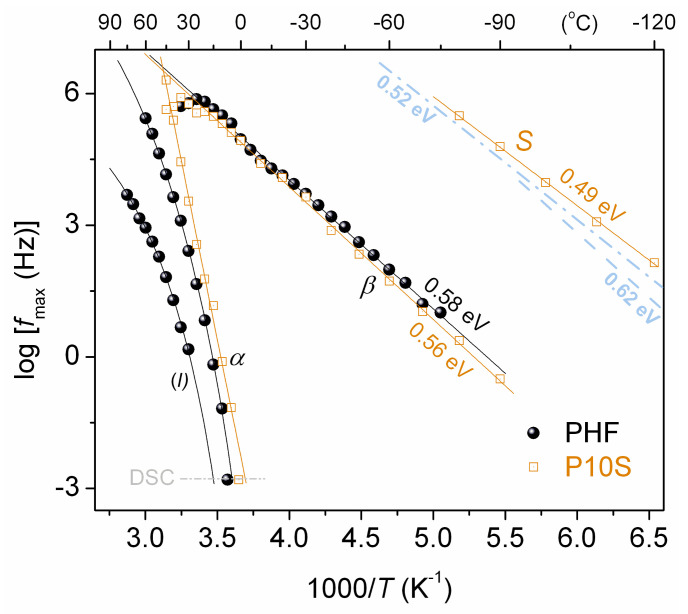
Dielectric relaxation map (Arrhenius plots) for PHF and P10S. The curved lines connecting the experimental points are fittings of the VTFH equation, whereas the straight lines are fittings of the Arrhenius equation. Included are the *E*_act_ values for the *β* and *S* relaxation. The added dashed/dotted lines correspond to data on a relaxation similar to our S here from previous studies on initial fumed silicas with specific surface area 166 m^2^/g (0.62 eV) [63] and 330 m^2^/g (0.52 eV) [65]. Included are data for the calorimetric *T*_g_ at the corresponding equivalent frequency.

**Figure 11:**
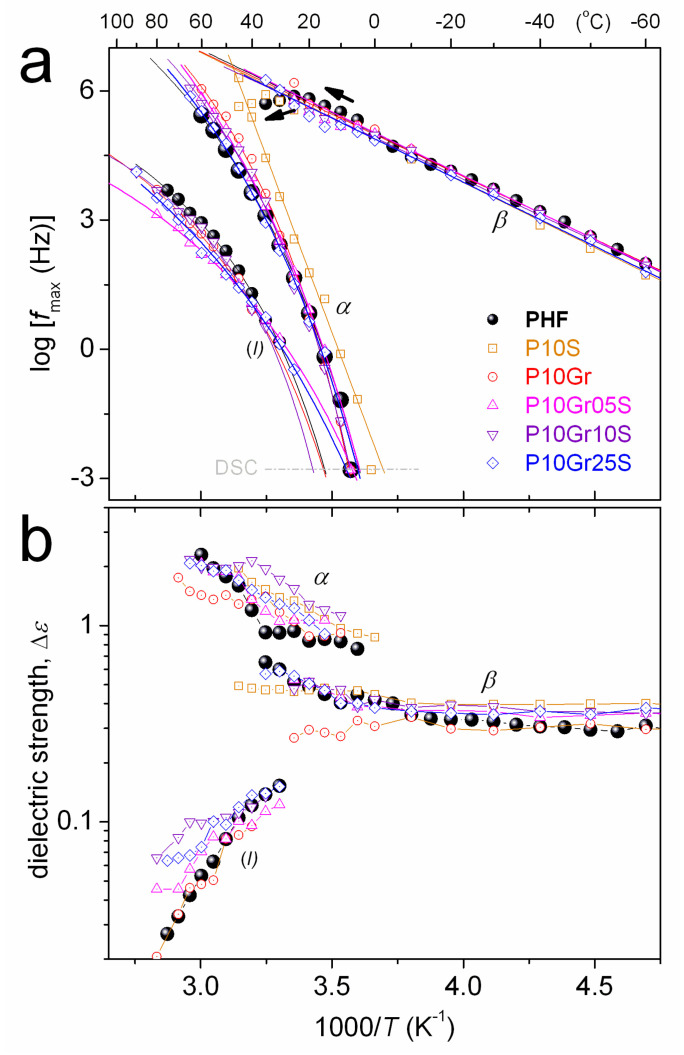
(**a**) Dielectric-calorimetric map (Arrhenius plots) and (**b**) reciprocal temperature dependence of dielectric strength, Δ*ε*, for all samples. The lines connecting the experimental points are fittings of the Arrhenius (straight lines) and the VTFH (curved lines). Included in (**a**) are data for the calorimetric *T*_g_ at the corresponding equivalent frequency. The added arrows in (**a**) mark the effects on the time scale of β when temperature increases to the region of *T*_g_.

The overall data for the frequency maxima of *ε΄΄*, *f*_max_, at all temperatures were inserted in common diagrams. Thus, the dielectric relaxation maps (Arrhenius plots) were constructed and are shown below in Figure 10 and Figure 11.

Figure 10 compares the time scale of the relaxation processes between PHF and P10S. The time scale as well as the *E*_act_ (~0.5 eV) of *S* process (*α*_HN_~0.2, *β*_HN_ = 1) correlates quite well with similar relaxations recorded in the past in initial silica nanoparticles with moderate amounts of hydration water (dashed/dotted lines in Figure 10) [63,64,65]. *S* relaxation, therein, was considered to arise from the surface hydroxyls of silica, most probably, with attached water molecules (–Si–OH···H_2_O) [66]. Therefore, we conclude to the similar origin of *S* in P10S here. Partial support to that can be extracted from the data by FTIR. In Figure 2a and in the wavenumber range around 3500 cm^–1^, the FTIR band arises from the presence of –OH and –NH_2_. In the corresponding FTIR band of P10S the absorbance is very poor (suppressed) suggesting the strong disturbance of –OH. This should be due to surface water (traces) on silica, rather than silica–PHF interaction as discussed previously.

*β* relaxation (*α*_HN_~0.3–0.4, *β*_HN_ = 1) is similar in the time scale (Figure 10 and Figure 11a) and in dielectric strength (Δ*ε*, Figure 11b) for all compositions. *β* exhibits a disturbance in time scale at elevated temperatures (arrows in Figure 11a). Similar observations were made in PPF- and PBF-based PNCs and in poly(ethylene furanoate) [67], with this disturbance on *β* seeming to correlate either with *T* approaching that of cold crystallization or the *T*_g_ [34,37]. More recently, we provided evidence for the second case in PPF-based PNCs [51]. In specific types of polymers, the phenomena of local (secondary) relaxations sensing the early glass transition stages have been rationalized in terms of the corresponding local polymer motions playing the role of precursors of the overall chain mobility [68,69,70].

**Figure 12:**
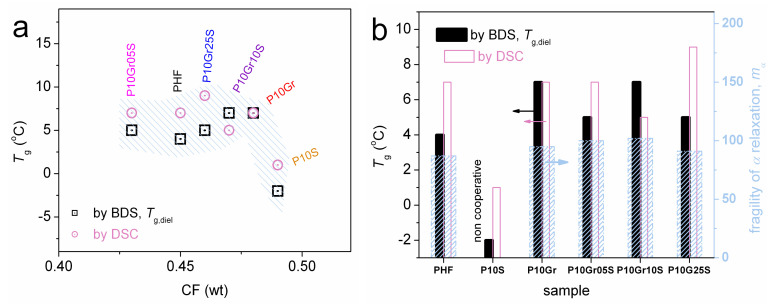
Dielectric against calorimetric *T*_g_ values shown (**a**) against CF and (**b**) comparatively with the fragility index *m*_α_ for all samples.

Similarly to the calorimetric glass transition in DSC, *α* relaxation in Figure 11a does not change in time scale from PHF to the most PNCs (except P10S). By extrapolating the time scale trends of *α* to the equivalent frequency of DSC, we estimate the so-called dielectric *T*_g_ point, *T*_g,diel_. *T*_g,diel_ is shown comparatively with *T*_g_ by DSC in Figure 12a. Therein, some trends or even values are in agreement between the two (in principle) different techniques. *α* is mainly symmetric (*α*_HN_~0.3–0.4, *β*_HN_ = 1), whereas in some cases (PHF, P10Gr, P10Gr05S) and only for the lower temperatures of recording the relaxation was fitted with an asymmetric HN term (*α*_HN_~0.6, *β*_HN_ = 0.4). The temperature evolution of Δ*ε* for *α* is demonstrated in Figure 11b. Δ*ε* increases with *T* for all samples, which is compatible with other cases of semicrystalline polymers with relatively high CF [71,72]. This increase in Δ*ε*(*T*) has been explained in terms of the gradual loosening of the constraints imposed on the amorphous chain diffusion by the crystals with *T*. The relatively large amount of crystallized polymer should also be the controlling factor for the quite similar time scale of *α* in Figure 11a, the latter being better understood also in Figure 12a which demonstrates the dependencies of *T*_g_ and *T*_g,diel_ on CF. Finally, we estimated the fragility, or cooperativity, index *m* for *α* relaxation following previous work [73]. The data of *m* are shown comparatively to *T*_g,diel_ and CF in the form of column diagrams in Figure 12b. The data between the three values do not show systematic changes. Nevertheless, considering the role of the high CF along with the data shown previously for *T*_c_ (Figure 5a), *T*_m_ (Figure 5b), and the XRD spectra (Figure 6a), we should see a complex situation in the PNCs that involves not only changes in the amount but also in the size and distribution of crystals (semicrystalline morphology).

Focusing on the exceptional behavior of P10S in Figure 11a and Figure 12a, *α* is significantly faster as compared to rest of the samples and, surprisingly, does not follow the VTFH law. On the contrary, for this sample the *α* exhibits a linear time scale (Arrhenius behavior), with severely suppressed cooperativity. The results indicate that the mobile amorphous PHF chains in P10S suffer strong spatial confinement [74,75]. Obviously, silica itself should not play the role of the confining mean. Thus, it is most possible that the confinement is due to the crystals or to be more precise, due to the severely altered semicrystalline morphology in P10S as compared to PHF and the other PNCs. From the data of this work we, unfortunately, are not able to prove the said confinement. POM measurements [19,60] in a future work could illuminate this point.

*Process I* is recorded slower than *α* by 1.5–2.5 orders of frequency magnitude. It is a symmetric process, with *α*_HN_~0.6, *β*_HN_ = 1 (Equation (2)), obeying the VTFH law (Figure 11a). The extrapolation of the VTFH curves of *process I* to the lower frequencies meet the calorimetric *T*_g_ range. We recall that the relaxation is observed in all PNCs except P10S and in neat PHF. Therefore, it should not arise from the filler. In PPF of low molecular weight (MW 10–20 kg/mol) [51] we recently recorded a similar relaxation exhibiting, however, larger magnitude (Δ*ε*) as compared to our case here. In that study [51], we concluded that the relaxation arises from the fluctuation of the polymer chain end-to-end vector (parallel to the PPF backbone), and moreover is the case of the so-called normal mode (NM) relaxation [76,77,78]. The MW of PHF here is expected quite low (~10k based on the intrinsic viscosity, Table 1), and does not change significantly in the PNCs. Consequently, *process I*, although weak, could possibly be a NM relaxation.

In previous work by many groups, results for semicrystalline PNCs have been evaluated in terms of the rigid amorphous fractions due to the crystals and the fillers (interfacial polymer fractions) [39,79,80,81,82,83,84,85,86,87,88]. Such evaluations could not be performed here, as we lack the information for the calorimetric/dielectric strength of glass transition (Δ*c*_p_, Δ*ε*) of the fully amorphous PHF and PNCs. As mentioned in a previous section, we could not produce amorphous PHF by conventional techniques. However, special techniques such as fast scanning calorimetry [87,88] are expected to succeed in eliminating crystallization. In addition to the already proposed POM measurements for the semicrystalline morphology, another point worth checking in the future is the quality of the nanofiller dispersion by direct microcopy techniques, e.g., transmission electron microscopy (TEM).

## 4. Conclusions

We studied the impact of graphene nanoplatelets and silica nanoparticles, individually and in combination, on the crystallization and molecular dynamics of PHF. For the latter we showed, for the first time, a molecular dynamics map. The crystallization of neat PHF was found to be faster (*T*_c_ = 110 °C in PHF, increased up to 123 °C in the PNCs) and stronger (CF~45 wt %) as compared to its homologues PPF and PBF. At low filler loadings and for the in situ PNCs synthesis, both graphene and silica were found to facilitate crystallization of PHF, mainly in the rate. Thus, it is concluded that these fillers act as additional crystallization nuclei. However, the impact of graphene on enhancing crystallization was found stronger compared to silica, which can be understood in terms of the larger AR of the platelets than that of spherical nanoparticles. The overall results by DSC and XRD suggest possible alternations in the semicrystalline morphology between the unfilled PHF and the PNCs. This was also partly reflected on the glass transition (*T*_g_~5–9 °C) and segmental dynamics (*α* relaxation, *T*_g,diel_). Dielectric and calorimetric *T*_g_ exhibited weak changes, ruled mainly by crystallinity (CF 45–49 wt %). Moreover, in the case of PHF filled uniquely with silica (1 wt %, P10S), *α* relaxation was recorded as significantly faster (*T*_g_ = 1 °C) and, surprisingly, showed absence of cooperativity. This result was explained in terms of severe spatial confinement imposed on the amorphous polymer chains by the crystals, most probably in combination with a special semicrystalline morphology. Regarding local dynamics, the time scale and strength of *β* relaxation of PHF was found unaffected in the PNCs, demonstrating a sensitivity in sensing large-scale changes in the matrix (*T*_g_). In P10S at quite low temperatures, the *S* relaxation was recorded, a process screening the dynamics of hydrated surface silanols of silica. Finally, at temperatures above *T*_g_ an additional relaxation was recorded in the PNCs and PHF, which show similarities to normal mode processes, i.e., the dielectric relaxation of the overall chain end-to-end dipole moment. Apart from showing the reach dynamics view, accompanied by interesting phenomena, we have also raised some questions in this work. These questions refer mainly to the situation in the semicrystalline morphology for which we gained only indirect indications. Measurements with more direct evidence (POM) in the future may shed further light on these issues. 

## Figures and Tables

**Table 1:** Description of the samples under investigation, code naming and characteristic values: intrinsic viscosity, [*n*], glass transition temperature, *T*_g_, crystallization temperature, *T*_c_, melting temperature, *T*_m1/2_, and crystalline fraction, CF.

Samples	Code Names	[*n*] (g/dL)	*T*_g_ (°C) (± δ*T*_g_)	*T*_c_ (°C)	*T*_m1/2_ (°C)	CF (wt) (± 0.01)
PHF	PHF	0.29	7 (± 1)	110	126/146	0.45
PHF + 1.0 wt % SiO_2_	P10S	0.23	1 (±2 )	114	127/144	0.49
PHF + 1.0 wt % graphene	P10Gr	0.25	7 (± 1)	121	140/148	0.48
PHF + 1.0 wt % graphene + 0.5 wt % SiO_2_	P10Gr05S	0.24	7 (± 1)	121	140/147	0.43
PHF +1.0 wt % graphene + 1.0 wt % SiO_2_	P10Gr10S	0.23	5 (± 1)	123	141/147	0.47
PHF + 1.0 wt % graphene + 2.5 wt % SiO_2_	P10Gr25S	0.25	9 (± 1)	116	140/148	0.46
